# Impact of Sarcopenia on Elderly Patients Undergoing Endoscopic Resection: Scoping Review

**DOI:** 10.1111/den.70210

**Published:** 2026-06-19

**Authors:** Hiroyuki Hisada, Hiroki Asano, Yosuke Tsuji, Dai Kubota, Yuko Miura, Hiroya Mizutani, Daisuke Ohki, Chihiro Takeuchi, Seiichi Yakabi, Keiko Niimi, Naomi Kakushima, Nobutake Yamamichi, Mitsuhiro Fujishiro

**Affiliations:** ^1^ Department of Gastroenterology, Graduate School of Medicine The University of Tokyo Tokyo Japan; ^2^ Department of Endoscopy and Endoscopic Surgery, Graduate School of Medicine The University of Tokyo Tokyo Japan; ^3^ Next‐Generation Endoscopic Computer Vision, Graduate School of Medicine The University of Tokyo Tokyo Japan

**Keywords:** endoscopic submucosal dissection, frailty, gastrointestinal neoplasms, prognosis, sarcopenia

## Abstract

**Objectives:**

Sarcopenia is a recognized risk factor in surgical oncology, yet its clinical significance in patients undergoing endoscopic resection (ER) remains controversial. This scoping review investigated the prevalence of sarcopenia and its association with technical outcomes, systemic complications, and long‐term prognosis in patients undergoing ER.

**Methods:**

We followed the PRISMA‐ScR framework and searched MEDLINE, Cochrane Central, Web of Science, and Ichushi Web up to November 12, 2025. Eligible studies involved patients undergoing ER (endoscopic submucosal dissection or endoscopic mucosal resection) for esophageal, gastric, or colorectal neoplasms, regardless of diagnostic criteria. A narrative synthesis was performed.

**Results:**

Twenty‐eight studies, predominantly retrospective cohorts from East Asia, were included. Prevalence ranged from 13.9% to 72.7%. Most studies defined sarcopenia by skeletal muscle mass on computed tomography; only one prospective study incorporated muscle strength. Sarcopenia was generally not associated with technical outcomes such as en bloc resection or perforation. However, it was associated with systemic complications: post‐procedural pneumonia was more frequent in two of three studies (odds ratio 3.16 in one), moderate‐to‐severe adverse events (CTCAE ≥ 2) in three of four, and hospital stay was prolonged in all three reporting studies. Sarcopenia was also associated with poor overall survival (hazard ratios 1.7–15.0), driven by non‐disease‐specific mortality.

**Conclusions:**

Sarcopenia does not compromise the technical safety of ER but is a critical marker for systemic complications and poor prognosis. Current evidence is limited by retrospective designs lacking muscle strength data. Future research should prioritize prospective assessment using multifaceted diagnostic criteria to refine risk stratification.

## 1 Introduction

With the rapid aging of the global population, the number of elderly patients diagnosed with early‐stage gastrointestinal cancer is increasing [1]. Endoscopic resection (ER) such as endoscopic submucosal dissection (ESD) and endoscopic mucosal resection (EMR) have become standard treatments for these lesions [2, 3]. Since these procedures are minimally invasive and allow for organ preservation, they are frequently indicated for elderly patients who may not tolerate radical surgery due to comorbidities or limited physiological reserve. However, chronological age alone is often an unreliable basis for determining the indication for ER.

Recent international guidelines, including European working group on sarcopenia in older people (EWGSOP)2, Asian working group for sarcopenia (AWGS)2019 and AWGS2025, have redefined sarcopenia beyond simple muscle loss [4, 5, 6]. The latest consensus underscores that skeletal muscle functions as an endocrine organ secreting myokines, influencing systemic metabolism and age‐related conditions ranging from frailty to cognitive decline. Thus, sarcopenia serves as a critical marker of biological aging. In the field of surgical oncology, abundant evidence indicates that sarcopenia is a robust predictor of postoperative complications, such as anastomotic leakage and poor long‐term survival [7]. Consequently, preoperative assessment of sarcopenia has become routine in risk stratification for gastrointestinal surgery.

In contrast, the clinical significance of sarcopenia in patients undergoing ER remains controversial [8, 9]. Recently, a systematic review and meta‐analysis synthesized available data and reported associations between sarcopenia and adverse outcomes [8]. Their analysis demonstrated low statistical heterogeneity for technical outcomes such as delayed bleeding (*I* ^2^ = 0%, *p* = 0.76) and perforation (*I* ^2^ = 0%, *p* = 0.59), but substantial heterogeneity for clinically critical outcomes including post‐procedural pneumonia (*I* ^2^ = 40%) and moderate‐to‐severe complications graded as CTCAE ≥ 2 (*I* ^2^ = 76%). Furthermore, most included studies relied solely on skeletal muscle mass for diagnosis, failing to incorporate muscle strength or physical function as recommended by sarcopenia guidelines [4, 5, 6]. Consequently, a simple quantitative synthesis cannot fully address the methodological gaps or clarify the true impact of sarcopenia defined by modern multifaceted criteria.

Given these methodological gaps, we conducted a scoping review of the literature and clarified the current state of knowledge. Specifically, this review addresses the following clinical questions: (1) the reported prevalence of sarcopenia in patients undergoing ER and its diagnostic approaches; (2) whether sarcopenia compromises the technical safety of ER; (3) whether sarcopenia is associated with systemic complications such as post‐procedural pneumonia; and (4) whether sarcopenia predicts long‐term overall survival following ER. Additionally, we aimed to identify gaps in the current diagnostic approaches to guide future research directions.

## 2 Methods

### 2.1 Study Design and Search Strategy

This study was designed as a scoping review to investigate the impact of sarcopenia on ER. This study was performed in accordance with the Preferred Reporting Items for Systematic Reviews and Meta‐Analyses extension for Scoping Reviews (PRISMA‐ScR) guidelines [10]. The PRISMA checklist is available as Appendix S1. A systematic literature search was conducted on November 12, 2025. We searched MEDLINE (PubMed), the Cochrane Central Register of Controlled Trials (CENTRAL), Web of Science, and Ichushi Web. Ichushi Web is a comprehensive bibliographic database of medical literature published in Japan, which was included to capture region‐specific evidence given the high prevalence of ER procedures in this country. The search strategy employed a combination of Medical Subject Headings (MeSH) and free‐text keywords using Boolean operators. Search terms for sarcopenia included “sarcopenia,” “muscular atrophy,” and specific metrics like “low muscle mass” or “skeletal muscle index.” These were combined with terms for ER, such as “endoscopic submucosal dissection” and “endoscopic mucosal resection.” The full search strategy for MEDLINE is detailed in Appendix S2.

### 2.2 Study Selection

We imported all identified records into Rayyan (Rayyan Systems Inc., Cambridge, MA, USA) to facilitate the screening process. Two reviewers (HH and HA) independently screened titles and abstracts to identify potentially relevant studies. Subsequently, full‐text articles were retrieved and assessed for eligibility. Disagreements at any stage were resolved through discussion between the two reviewers.

### 2.3 Eligibility Criteria

Eligibility criteria were established based on the Population, Concept, and Context framework. The review included patients who underwent ER, specifically ESD or EMR, for esophageal, gastric, or colorectal neoplasms. Regarding the concept of interest, we aimed to comprehensively map the evidence in this emerging field; therefore, we did not limit inclusion to specific diagnostic criteria for sarcopenia, such as those established by AWGS 2025 or EWGSOP 2. We included studies defining sarcopenia based on skeletal muscle mass alone measured via computed tomography using metrics such as the L3 skeletal muscle index or psoas muscle index, or via bioelectrical impedance analysis (BIA), as well as those relying on unique institutional definitions. No restrictions were placed on the context, such as the country or type of healthcare facility. We included original articles, comprising prospective and retrospective cohort studies, case series, and conference abstracts. Given the high volume of relevant literature from Japan, no language restrictions were applied to ensure a comprehensive review. Additionally, studies focusing on non‐resection endoscopic procedures, such as endoscopic retrograde cholangiopancreatography or percutaneous endoscopic gastrostomy, were excluded.

### 2.4 Data Charting and Data Items

Data extraction was performed using a standardized form developed for this study. We collected the following study characteristics: first author, year of publication, and country. Regarding the concept of interest, we recorded the specific definition of sarcopenia used in each study and its reported prevalence. We also abstracted data on clinical outcomes associated with ER.

### 2.5 Synthesis of Results

Given the anticipated heterogeneity in study designs and sarcopenia definitions, we did not perform a meta‐analysis. Instead, we conducted a narrative synthesis of the findings. We summarized the study characteristics, the reported prevalence of sarcopenia, and its impact on treatment outcomes in tabular format to facilitate a descriptive comparison.

## 3 Results

### 3.1 Selection of Sources of Evidence

**FIGURE 1:**
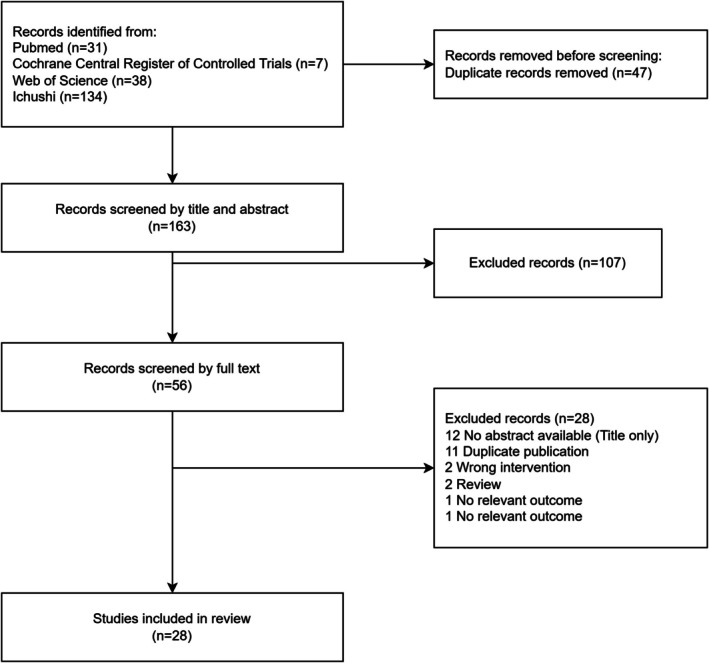
PRISMA flow diagram illustrates the literature search and study selection process. The flow diagram depicts the identification, screening, eligibility assessment, and final inclusion of studies for the scoping review. Out of 210 identified records, 28 studies were ultimately included.

A total of 210 records were identified through database searching (PubMed: 31, CENTRAL: 7, Web of Science: 38, and Ichushi‐Web: 134). After removing duplicates, 163 records remained for screening. Based on title and abstract screening, 107 records were excluded. Fifty‐six full‐text articles were retrieved and assessed for eligibility. Of these, 28 were excluded mainly due to the unavailability of full text (*n* = 12) and duplicate publications (*n* = 11), as well as wrong interventions or study designs. Consequently, 28 studies were included in this scoping review. The study selection process is illustrated in the PRISMA flow diagram (Figure 1).

### 3.2 Characteristics of Sources of Evidence

The 28 included studies were published between 2018 and 2025, with an increase in reports observed from 2022 onward. These consisted of 16 original articles and 12 conference abstracts. Geographically, the majority of reports originated from Japan (*n* = 23, 82%), with the remainder from South Korea. The sample sizes of the included studies ranged from 20 to 3289 patients. The reported median or mean ages of the study populations ranged from 62.9 to 86 years. Regarding age criteria, 15 of the 28 studies did not impose strict age limitations. Among the studies that restricted their cohorts to older patients, a cutoff age of 65 years was the most frequently used [11, 12, 13, 14, 15, 16]. In terms of study design, nearly all were retrospective cohort studies (*n* = 27), with only one prospective study. In terms of target organs for ER, gastric ESD was the most predominant subject (*n* = 19), followed by esophageal (*n* = 7) and colorectal (*n* = 2) ESD (Table 1).

**TABLE 1:** Characteristics of the included studies.

Author (year)	Country	Publication type	Study design	Target organ	Age	No. of participants	Sarcopenia (%)	Definition of sarcopenia
Choi et al. (2018) [17]	Korea	Original article	Retrospective	Gastric	62.9*	302	30.6%	CT (L3 SMI: < 5 2.4 cm^2^/m^2^ for men, < 38.5 cm^2^/m^2^ for women)
Kubota et al. (2019) [18]	Japan	Conference abstract	Retrospective	Gastric	69.6*	71	57.7%	CT (L3 PMI: < 6.36 cm^2^/m^2^ for men, < 3.92 cm^2^/m^2^ for women)
Yamamoto et al. (2020) [19]	Japan	Conference abstract	Retrospective	Gastric	79.7†	55	46.4%	CT (L3 PMI: < 6.0 cm^2^/m^2^ for men, < 3.4 cm^2^/m^2^ for women)
Arao et al. (2021) [20]	Japan	Original article	Retrospective	Gastric	83†	157	42.0%	CT (L3 SMI: < 42.0 cm^2^/m^2^ for men, < 38.0 cm^2^/m^2^ for women)
Goto et al. (2021) [21]	Japan	Original article	Retrospective	Colorectal	71.3*	244	49.6%	CT (L3 SMI: < 50.0 cm^2^/m^2^ for men, < 39.0 cm^2^/m^2^ for women)
Kim et al. (2021) [22]	Korea	Original article	Retrospective	Gastric	82†	280	61.8%	CT (L3 SMI: < 52.4 cm^2^/m^2^ for men, < 38.5 cm^2^/m^2^ for women)
Suzuki et al. (2021) [23]	Japan	Original article	Retrospective	Esophageal	69†	241	56.4%	CT (L3 PMI: < 5.68 cm^2^/m^2^ for men, < 4.16 cm^2^/m^2^ for women)
Hisada et al. (2022) [24]	Japan	Original article	Retrospective	Gastric	76†	700	34.1%	CT (L3 SMI: < 40.8 cm^2^/m^2^ for men, < 34.9 cm^2^/m^2^ for women)
Hisada et al. (2022) [14]	Japan	Original article	Retrospective	Gastric	77†	767	14.2%	CT (SMI: < 42.0 for men, < 38.0 for women; IMAC: > −0.31 for men, > −0.19 for women)
Ito et al. (2022) [25]	Japan	Original article	Retrospective	Gastric	82†	88	72.7%	CT (L3 PMI: < 6.36 cm^2^/m^2^ for men, < 3.92 cm^2^/m^2^ for women)
Kudo et al. (2022) [26]	Japan	Original article	Retrospective	Esophageal	66†	75	30.7%	CT (L3 PMI: < 6.0 cm^2^/m^2^ for men, < 3.4 cm^2^/m^2^ for women)
Ogata et al. (2022) [27]	Japan	Original article	Retrospective	Gastric	71†	1439	NR	CT (L3 PMI: < 6.36 cm^2^/m^2^ for men, < 3.92 cm^2^/m^2^ for women)
Saiki et al. (2022) [28]	Japan	Conference abstract	Retrospective	Gastric	84†	56	21.4%	CT (L3 PMI: Not reported)
Yamamoto et al. (2022) [29]	Japan	Conference abstract	Retrospective	Gastric	NR	55	NR	CT (L3 PMI: Not reported)
Kim et al. (2023) [12]	Korea	Original article	Retrospective	Gastric	73*	3289	47.8%	CT (L3 SMI: < 53.4 cm^2^/m^2^ for men, < 38.5 cm^2^/m^2^ for women)
Inuyama et al. (2023) [16]	Japan	Conference abstract	Retrospective	Gastric	78†	24	NR	BIA (SMI: Not reported)
Matsuno et al. (2023) [13]	Japan	Conference abstract	Retrospective	Esophageal	NR	38	NR	CT (L3 SMI: Not reported)
Nakao et al. (2023) [30]	Japan	Conference abstract	Retrospective	Esophageal	69†	20	NR	CT (L3 PMI: Not reported)
Shiraishi et al. (2023) [31]	Japan	Conference abstract	Retrospective	Gastric	86†	35	NR	CT (L3 PMI: < 4.04 cm^2^/m^2^ for men, < 3.02 cm^2^/m^2^ for women)
Hisada et al. (2024) [15]	Japan	Original article	Retrospective	Colorectal	75†	497	13.9%	CT (SMI: < 42.0 for men, < 38.0 for women; IMAC: > −0.28 for men, > −0.12 for women)
Tanaka et al. (2024) [32]	Japan	Original article	Retrospective	Gastric	80†	263	24.3%	Original (SARC‐F + EBM score ≥ 12)
Taniguchi et al. (2024) [11]	Japan	Original article	Prospective	Esophageal	77.6*	20	25.0%	AWGS2019
Arai et al. (2024) [33]	Japan	Conference abstract	Retrospective	Gastric	73.4*	83	22.0%	CT (L3 PMI: < 6.0 cm^2^/m^2^ for men, < 3.4 cm^2^/m^2^ for women)
Hayamizu et al. (2024) [34]	Japan	Conference abstract	Retrospective	Gastric	NR	64	26.6%	CT (L3 PMI: < 6.0 cm^2^/m^2^ for men, < 3.4 cm^2^/m^2^ for women)
Kim et al. (2025) [35]	Korea	Conference abstract	Retrospective	Gastric	NR	329	NR	CT (L3 SMII: Not reported)
Kim et al. (2025) [36]	Korea	Original article	Retrospective	Gastric	65†	2267	NR	CT (L3 SMI: < 39.33 for men, < 27.77 for women)
Tanno et al. (2025) [37]	Japan	Original article	Retrospective	Esophageal	70†	450	NR	CT (L3 SMI: < 42.0 for men, < 38.0 for women; PMI: < 6.36 cm^2^/m^2^ for men, < 3.92 cm^2^/m^2^ for women; IMAC: > −0.358 for men, > −0.229 for women)
Yumoto et al. (2025) [38]	Japan	Conference abstract	Retrospective	Esophageal	NR	191	49.2%	BIA (SMI: < 7.0 cm^2^/m^2^ for men, < 5.7 cm^2^/m^2^ for women)

Abbreviations: AWGS, Asian Working Group for Sarcopenia; BIA, bioelectrical impedance analysis; CT, computed tomography; IMAC, intramuscular adipose tissue content; L3, third lumbar vertebra; NR, not reported; PMI, psoas muscle mass index; SMI, skeletal muscle mass index.

* Mean.

^†^ Median.

### 3.3 Diagnostic Criteria and Prevalence Diagnostic Criteria for Sarcopenia

**FIGURE 2:**
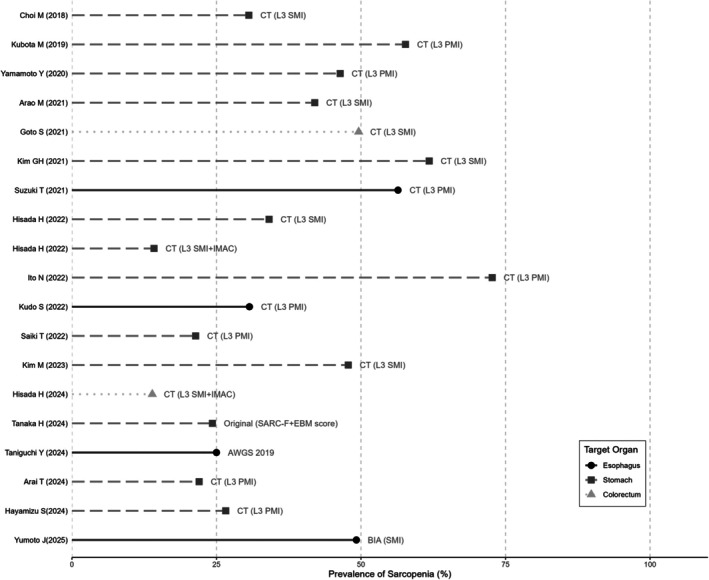
Prevalence of sarcopenia by target organ and definition data points represents the reported prevalence of sarcopenia in each study. Markers distinguish the target organs: circles for esophagus, squares for stomach, and triangles for colorectum. The diagnostic modality and specific indices used are indicated beside each data point. AWGS, Asian Working Group for Sarcopenia; BIA, bioelectrical impedance analysis; CT, computed tomography; IMAC, intramuscular adipose tissue content; L3, third lumbar vertebra; PMI, psoas muscle mass index; SMI, skeletal muscle mass index.

Concerning the diagnosis of sarcopenia, only one study applied the AWGS 2019 criteria; the majority defined sarcopenia based on skeletal muscle mass indices derived from CT images at the L3 level, such as SMI or PMI [6, 10, 11, 12, 13, 17, 18, 19, 20, 21, 22, 23, 24, 25, 26, 27, 28, 29, 30, 31, 33, 34, 35, 36]. However, the thresholds for these indices were inconsistent, as most studies used study‐specific median values or previously reported cutoffs instead of standardized guidelines (Table 1). A subset of these studies additionally incorporated the Intramuscular Adipose Content (IMAC) to assess muscle steatosis [14, 15, 37]. Based on these definitions, prevalence rates ranged from 14.2% to 72.7% for gastric ESD, 25.0%–56.4% for esophageal ESD, and 13.9%–49.6% for colorectal ESD (Figure 2). In the only prospective study that evaluated sarcopenia according to the AWGS 2019 criteria assessing both muscle strength and skeletal muscle mass, the prevalence was 25% [11].

### 3.4 Technical Safety and Procedure‐Related Outcomes

Table 2 summarizes the technical outcomes of ER. Technical safety and procedure‐related outcomes were generally comparable between patients with and without sarcopenia. En bloc resection rates exceeded 94% in both groups across all five reporting studies, with no significant differences observed. Similarly, curative resection rates (ranging from 73.9% to 85.9% in sarcopenia groups) were comparable in all six studies, and delayed bleeding (1.6%–11.0% in sarcopenia groups) was not significantly associated with sarcopenia in any of the seven reporting studies. Perforation rates remained low (0%–8.7% in sarcopenia groups) and were comparable across all eight studies. Procedure time showed mixed findings: Two out of seven studies reported significantly longer procedure time in the sarcopenia group, namely one gastric ESD cohort (150 min vs. 125 min, *p* < 0.001) [24] and one colorectal ESD cohort (100 min vs. 70 min, *p* = 0.004) [15], whereas the remaining five studies reported comparable procedure times. With regard to post‐electrocoagulation syndrome (PECS) in colorectal ESD, findings from two studies were inconsistent: one reported a lower unadjusted incidence in the sarcopenia group (0.7% vs. 5.1%, *p* = 0.04) that was not retained in subgroup analyses adjusting for tumor location and procedure time [21], while the other reported no significant difference (5.8% vs. 2.8%, *p* = 0.26) [15].

**TABLE 2:** Summary of technical outcomes and procedure‐related complications.

	Target	En bloc resection	Curative resection	Delayed bleeding	Perforation	PECS	Procedure time
Arao et al. (2021) [20]	Gastric	NR	NR	6.1% vs. 2.2% (*p* = 0.24)	1.5% vs. 0.0% (*p* = 0.42)	NR	56 min vs. 51 min (*p* = 0.40)
Goto et al. (2021) [21]	Colorectal	94.1% vs. 97.1% (*p* = 0.18)	77.6% vs. 81.8% (*p* = 0.24)	3.0% vs. 4.4% (*p* = 0.39)	4.5% vs. 6.6% (*p* = 0.31)	**0.7% vs 5.1% (*p* = 0.04)**	67.7 min vs. 64.6 min (*p* = 0.67)
Hisada et al. (2022) [24]	Gastric	100% vs. 100% (*p* = 1.00)	81% vs. 81% (*p* = 0.96)	8.0% vs. 5.0% (*p* = 0.08)	4.0% vs. 2.0% (*p* = 0.15)	NR	**150 min vs 125 min (*p* < 0.001)**
Hisada et al. (2022) [14]	Gastric	99.1% vs. 99.4% (*p* = 0.54)	83.5% vs. 81.5% (*p* = 0.48) *	11.0% vs. 7.5% (*p* = 0.20)	1.8% vs. 1.7% (*p* = 1.00)	NR	116 min vs. 120 min (*p* = 0.64)
Ito et al. (2022) [25]	Gastric	NR	86% vs. 67% (*p* = 0.07)	3.1% vs. 0% (*p* = 1.00)	3.1% vs. 4.2% (*p* = 1.00)	NR	NR
Kim et al. (2023) [12]	Gastric	97% vs. 97% (*p* = 0.45)	NR	NR	2% vs. 2% (*p* = 0.85)	NR	44 min vs. 44 min (*p* = 0.63)
Hisada et al. (2024) [15]	Colorectal	97.1% vs. 97.0% (*p* = 1.00)	73.9% vs. 78.8% (*p* = 0.36)	4.4% vs. 1.9% (*p* = 0.19)	8.7% vs. 3.7% (*p* = 0.06)	5.8% vs. 2.8% (*p* = 0.26)	**100 min vs. 70 min (*p* = 0.004)**
Tanaka et al. (2024) [32]	Gastric	100% vs. 99.0% (*p* = 1.00)	85.9% vs. 86.4% (*p* = 1.00)	1.6% vs. 2.5% (*p* = 1.00)	0% vs. 2.5% (*p* = 0.34)	NR	60 min vs. 60 min (*p* = 0.93)

*Note:* Bold values indicate statistically significant differences (*p* < 0.05). Values are presented as sarcopenia group versus non‐sarcopenia group.

Abbreviations: NR, not reported; NS, not significant; PECS, post‐electrocoagulation syndrome.

* Curative resection corresponds to eCuraA/B category; *p* value reflects the overall eCura category comparison.

### 3.5 Systemic and Long‐Term Impact

Table 3 presents the systemic and long‐term outcomes. Post‐procedural pneumonia was more frequent among patients with sarcopenia in two out of three studies [12, 20], with one study reporting an odds ratio of 3.16 for gastric ESD [20]. The remaining study (a prospective esophageal cancer cohort) showed a nonsignificant trend despite a higher incidence (60% vs. 20%) owing to small sample size [11]. For moderate‐to‐severe adverse events (CTCAE ≥ 2), findings were mixed: Three out of four studies reported a higher incidence in the sarcopenia group, whereas one study found comparable rates [14, 15, 24, 32]. Length of hospital stay was longer in all three studies of sarcopenia cohorts [15, 24, 32].

**TABLE 3:** Summary of systemic complications and long‐term outcomes.

	Target	Pneumonia	Physiological stress response	CTCAE grade 2+	Physical/Nutritional deterioration	Recurrence	Sarcopenia progression/Incidence	OS	Length of stay
Choiet al. (2018) [17]	Gastric	NR	NR	NR	NR	NS	NR	Shorter (*p* = 0.049)	NR
Kubota et al. (2019) [18]	Gastric	NR	NR	NR	NR	34.1% vs. 6.7% (4‐year, *p* < 0.01)	NR	NR	NR
Yamamoto et al. (2020) [19]	Gastric	NR	NR	NR	NR	NR	Prevalence Change (Pre → 3y): Observation: 59.2% → 66.7%. Additional surgery: 33.3% → 75.0%	NR	NR
Arao et al. (2021) [20]	Gastric	OR = 3.16 (1.18–8.50), *p* = 0.023	NR	NR	NR	NR	NR	NR	NR
Kim et al. (2021) [22]	Gastric	NR	NR	NR	NR	NR	NR	5‐year OS: 68.5% vs. 84.1% (*p* = 0.046), HR 1.27 (0.84–1.92), *p* = 0.266	NR
Suzuki et al. (2021) [23]	Esophageal	NR	NR	NR	NR	NR	NR	NS	NR
Hisada et al. (2022) [24]	Gastric	NR	NR	17% vs. 10% (*p* = 0.005), OR 1.79 (1.11–2.89), *p* = 0.016	NR	NR	NR	NR	8 days vs. 7 days (*p* < 0.001)
Hisada et al. (2022) [14]	Gastric	NR	NR	22% vs. 12.5% (*p* = 0.01), OR 1.90 (1.05–3.45), *p* = 0.03	NR	NR	NR	5‐year OS: 76.1% vs. 98.8%, HR 15.0 (5.82–38.5), *p* < 0.001	NS
Ito et al. (2022) [25]	Gastric	NR	NR	NR	NR	NR	NR	HR 2.89 (1.11–7.54), *p* = 0.03	NR
Kudo et al. (2022) [26]	Esophageal	NR	NS	NR	NR	NR	NR	NR	NR
Ogata et al. (2022) [27]	Gastric	NR	NR	NR	NR	NR	NR	Early (< 3 years): HR 1.70 (1.07–2.71), *p* = 0.024, Late (≥ 3 years): HR 1.31 (0.96–1.79), *p* = 0.089	NR
Saiki et al. (2022) [28]	Gastric	NR	NR	NR	ADL score: Lower (*p* < 0.05)	NR	NR	Lower trend (*p* < 0.1)	NR
Yamamoto et al. (2022) [29]	Gastric	NR	NR	NR	NR	NS	NR	Lower trend (*p* = 0.068)	NR
Kim et al. (2023) [12]	Gastric	OR = 2.30 (1.10–4.83), *p* = 0.028	NR	NR	NR	NR	NR	NR	NR
Inuyama et al. (2023) [16]	Gastric	NR	NR	NR	Leg strength: Lower	NR	NR	NR	NR
Matsuno et al. (2023) [13]	Esophageal	NR	NR	NR	Muscle Mass loss (> 10%): 16% of patients	NR	NR	NR	NR
Nakao et al. (2023) [30]	Esophageal	NR	NR	NR	PMI change: OPE: −11.4% (vs Obs/CRT: *p* = 0.012)	NR	NR	NR	NR
Shiraishi et al. (2023) [31]	Gastric	NR	NR	NR	NR	NR	NR	5.5 months vs. 36.0 months, *p* < 0.05	NR
Hisada et al. (2024) [15]	Colorectal	NR	NR	37.7% vs. 10.5% (*p* < 0.001), OR 3.78 (1.85–7.73), *p* < 0.001	NR	NR	NR	NS	7 days vs. 6 days (*p* < 0.001)
Tanaka et al. (2024) [32]	Gastric	NR	NR	NS	NR	NR	NR	NR	≧ 10 days: 12.5% vs. 3.5% (*p* = 0.012), OR 4.02 (1.32–12.2), *p* = 0.014
Taniguchi et al. (2024) [11]	Esophageal	60% vs. 20%, *p* = 0.11	NR	NR	NR	NR	NR	NR	NR
Arai et al. (2024) [33]	Gastric	NR	NS	NR	NR	NR	NR	NR	NR
Hayamizu et al. (2024) [34]	Gastric	NR	NS	NR	NR	NR	NR	NR	NR
Kim et al. (2025) [35]	Gastric	NR	NR	NR	NR	NR	EGD vs. Gastrectomy 3 years (Incidence): 40.6% vs. 25.7% (*p* < 0.01)	NR	NR
Kim et al. (2025) [36]	Gastric	NR	NR	NR	Muscle Mass Change:ESD vs. Surg (Diff: 3.2%, *p* < 0.001)	NR	EGD vs. Gastrectomy (Incidence): 1.9% vs. 3.4% (*p* = 0.139)	NR	NR
Tanno et al. (2025) [37]	Esophageal	NR	NR	NR	NR	NR	NR	Low SMI: HR 1.65 (1.06–2.56), *p* = 0.026, Late (≥ 3 years): Low SMI + High IMAC HR 4.53 (1.72–11.95), *p* = 0.002	NR
Yumoto et al. (2025) [38]	Esophageal	NR	NR	NR	NR	NR	NR	3‐year OS: 91.7% vs. 96.0%, *p* = 0.303	NR

Abbreviations: ADL, activities of daily living; CTCAE, Common Terminology Criteria for Adverse Events; HR, hazard ratio; NR, not reported; NS, not significant; OR, odds ratio; OS, overall survival.

Overall, regarding long‐term outcomes, sarcopenia was generally associated with worse OS across the included reports, although a few studies showed no clear association [14, 17, 25, 27, 28, 29, 31, 37]. Where hazard ratios were available, the magnitude was mostly modest to moderate, with notable heterogeneity: one gastric ESD cohort reported a markedly higher risk (HR 15.0; 95% CI 5.82–38.5), another cohort reported HR 2.89 (1.11–7.54). A third gastric ESD cohort showed a time‐dependent pattern, with a stronger association in the early period (< 3 years: HR 1.70 [1.07–2.71]) but no statistically significant association at ≥ 3 years (HR 1.31 [0.96–1.79]) [14, 25, 27].

Regarding specific causes of death, non‐disease‐specific mortality accounted for the majority of deaths. In patients undergoing gastric ESD, malignancies of other organs and pneumonia were the leading causes of death, whereas disease‐specific mortality was infrequent [14, 25, 27]. Similarly, in the esophageal ESD cohort, other primary malignancies, such as head and neck or lung carcinoma, were the most common causes of mortality [37].

Concerning the physiological stress response, no studies reported an increase associated with sarcopenia [26, 33, 34]. While the risk of developing new‐onset sarcopenia after ESD was reported to be lower compared to surgical resection, a decline in skeletal muscle mass and activities of daily living (ADL) following ESD was documented in several studies [16, 28, 35, 36].

## 4 Discussion

This scoping review summarized the current evidence regarding sarcopenia in patients undergoing ER. The prevalence of sarcopenia ranged widely from 13.9% to 72.7%, depending on the target organ and diagnostic criteria. Despite the high prevalence, the synthesis of results indicated that sarcopenia was not associated with technical outcomes, such as en bloc resection or procedure‐related complications. In contrast, it was identified as a negative prognostic factor for systemic complications, particularly pneumonia, and OS.

The included studies demonstrated considerable heterogeneity in patient age, with median or mean ages ranging from 62.9 to 86 years. The lack of age restrictions in several cohorts likely contributes to the wide range of reported sarcopenia prevalence and introduces variability in endoscopy‐related outcomes. Nevertheless, this broader age inclusion aligns with current international guidelines. EWGSOP2 removed strict age limitations to address secondary sarcopenia in younger adults, and the AWGS2025 expanded its target population to include individuals aged 50–64 years for early intervention [4, 6]. Because gastrointestinal neoplasms indicated for ESD frequently affect middle‐aged patients, assessing sarcopenia across a wider age spectrum is clinically relevant.

While the broad age inclusion is clinically justified, a more critical issue in interpreting the current literature is the diagnostic approach adopted in most studies. Specifically, the majority relied solely on skeletal muscle mass, largely due to their retrospective design. Standard international guidelines, such as the AWGS2019 and EWGSOP2, define sarcopenia based on a combination of loss of skeletal muscle mass and reduced muscle strength or physical function [4, 5]. Notably, only one prospective study by Taniguchi et al. incorporated muscle strength and physical function into the diagnostic criteria in accordance with AWGS2019 [11]. The recent AWGS2025 update defines sarcopenia based on low muscle mass and reduced muscle strength. Consequently, physical performance measures such as gait speed are now reserved for evaluating clinical outcomes rather than serving as a diagnostic component [6]. Likewise, EWGSOP2 guidelines prioritize muscle strength as the primary indicator of sarcopenia [4]. Taken together, relying exclusively on CT‐based muscle mass assessment likely leads to an underestimation of the true burden of sarcopenia. Beyond strength, muscle quality is an additional dimension often overlooked by simple mass measurements. EWGSOP2 guidelines recognize myosteatosis, which is defined as the infiltration of fat into muscle, as a key determinant of muscle quality [4]. High IMAC, a marker of myosteatosis, has been reported as a significant predictor of early mortality in other clinical settings, such as liver transplantation [39]. In the present review, a few studies incorporated IMAC to define sarcopenia as a measure of muscle quality [14, 15, 37]. Future studies should systematically integrate strength testing such as grip strength, physical performance assessments including chair rise or gait speed, and quality metrics like IMAC or radiomics‐derived texture features to refine risk stratification for ER.

The results of the present review suggest that sarcopenia does not compromise the technical success of ER. Unlike surgical resection, where sarcopenia has been demonstrated to be a significant risk factor for anastomotic leakage and impaired wound healing, the technical outcomes of ER are largely determined by local factors [40]. Previous studies have reported that en bloc resection rates and perforation rates depend on multiple factors. These include lesion‐related factors such as tumor size, submucosal fibrosis, and deep invasion, as well as procedural factors including poor maneuverability and the endoscopist's experience [41, 42]. Consequently, these local and technical determinants exert a stronger influence than sarcopenia. Our review identified that sarcopenia does not amplify the physiological stress response or energy metabolism changes following ESD. While major surgical interventions often induce a hypercatabolic state that demands substantial physiological reserves, the surgical stress associated with ESD is relatively mild [43]. Thus, even patients with depleted muscle mass appear to possess sufficient metabolic reserve to tolerate the procedure without significant adverse physiological reactions.

In contrast to preserved technical safety, regarding systemic complications, several studies have identified sarcopenia as a significant risk factor for post‐procedural pneumonia following gastric ESD [12, 20]. The increased risk observed in these studies is likely attributable to sarcopenic dysphagia, which is characterized by the atrophy of swallowing muscles [44]. Supporting this mechanism, Taniguchi et al. demonstrated that patients with sarcopenia exhibited significantly lower preoperative tongue pressure, as well as reduced pharyngeal and upper esophageal pressures, compared to the non‐sarcopenia group [11]. These specific functional impairments suggest that compromised swallowing mechanics directly contribute to the increased risk of post‐procedural pneumonia. Furthermore, reduced respiratory muscle strength may impair the coughing reflex, further increasing susceptibility to aspiration during or after sedation.

Regarding long‐term outcomes, the majority of reports in this review identified sarcopenia as a negative prognostic factor for OS, despite the technical success of the procedure. Interestingly, non‐disease‐specific mortality, such as pneumonia or malignancies of other organs, was far more frequent in these patients, whereas disease‐specific mortality was infrequent [14, 25, 27]. Furthermore, although ESD is less invasive than surgery, several studies documented a decline in skeletal muscle mass and ADL following the procedure [16, 28, 35, 36]. However, given the relatively low invasiveness of ESD itself, this functional decline likely reflects the underlying trajectory of sarcopenic patients rather than a direct consequence of the procedure. Indeed, the minimally invasive nature of ER allows treatment to be offered even to patients with limited physiological reserve in whom long‐term survival may not be substantially improved by curative resection. Consequently, in patients with severe sarcopenia, the survival benefit of curative ER may be offset by the high risk of death from non‐cancer causes.

There are some limitations in this scoping review. First, significant heterogeneity existed regarding the diagnostic criteria and cutoff values for sarcopenia across the included studies. This variability precludes a direct comparison of prevalence rates and outcomes, highlighting the need for a standardized consensus adherent to established guidelines, such as AWGS2025. Second, nearly all included studies were retrospective and limited to patients who actually underwent ESD; the most vulnerable patients with severe sarcopenia or frailty may have been excluded from treatment due to poor performance status, potentially leading to selection bias and underestimation of the true burden of sarcopenia. Third, the majority of the studies originated from East Asia, particularly Japan and South Korea. Since body composition and the prevalence of sarcopenia differ across ethnicities, these findings may not be fully generalizable to Western populations. Fourth, 12 out of 28 studies (42.9%) included in this review were conference abstracts. While including gray literature from databases was necessary to capture recent and region‐specific evidence, these reports often lack the rigorous peer‐review process of full‐text journal articles. Furthermore, the limited methodological details provided in these abstracts may restrict the depth of the data synthesis. Finally, we did not perform a quantitative meta‐analysis, as a recent meta‐analysis by Su et al. has already pooled the largely overlapping evidence base [8]. A future meta‐analysis will be warranted once prospective studies using guideline‐concordant multifaceted diagnostic criteria become available.

In conclusion, sarcopenia is highly prevalent among patients undergoing ER but does not hinder the technical success of the procedure. However, it serves as a critical predictor of systemic complications, such as pneumonia, and poor long‐term survival driven by non‐cancer causes. Current diagnostic practices relying solely on muscle mass in retrospective settings are insufficient to capture the true frailty of these patients.

To bridge these evidence gaps, future research should prioritize prospective studies that assess muscle strength, a core diagnostic component in updated guidelines such as AWGS2025, in addition to muscle mass. Furthermore, clinical strategies must evolve from lesion‐oriented treatment to patient‐oriented management. Supporting this approach, in the field of hepatic surgery, a randomized clinical trial demonstrated that a 6‐week preoperative program combining physical exercise and nutritional support significantly reduced postoperative morbidity in patients with sarcopenia [45]. It remains to be elucidated whether similar multimodal interventions can improve systemic outcomes, such as pneumonia prevention and long‐term survival, in patients undergoing ER.

## Author Contributions

All authors contributed substantially to the study's conception and design. Data collection, extraction, and synthesis were performed by Hiroyuki Hisada and Hiroki Asano. Hiroyuki Hisada drafted the initial manuscript, and all authors revised it critically for important intellectual content. All authors approved the final version of the manuscript and agree to be accountable for all aspects of the work to ensure the accuracy and integrity of any part of the study.

## Funding

The authors have nothing to report.

## Ethics Statement

The authors have nothing to report.

## Consent

The authors have nothing to report.

## Conflicts of Interest

Yosuke Tsuji and Hiroya Mizutani are affiliated with an endowed chair by AI Medical Service Inc., which is not relevant to this research. Naomi Kakushima is a deputy editor in chief.

## Supporting information


**Appendix S1:** PRISMA‐ScR checklist.
**Appendix S2:** Search strategy.
